# System-Wide Thromboprophylaxis Interventions for Hospitalized Patients at Risk of Venous Thromboembolism: Focus on Cross-Platform Clinical Decision Support

**DOI:** 10.3390/jcm13072133

**Published:** 2024-04-07

**Authors:** Nikolaos Tsaftaridis, Mark Goldin, Alex C. Spyropoulos

**Affiliations:** 1 Institute of Health System Science, Feinstein Institutes for Medical Research, Northwell Health, Manhasset, NY 11030, USA; ntsaftaridis@northwell.edu (N.T.); mgoldin@northwell.edu (M.G.); 2 Anticoagulation and Clinical Thrombosis Services, Northwell Health at Lenox Hill Hospital, New York, NY 10075, USA; 3 The Donald and Barbara Zucker School of Medicine at Hofstra/Northwell, Hempstead, NY 11549, USA; 4 Elmezzi Graduate School of Molecular Medicine, Manhasset, NY 11030, USA

**Keywords:** venous thromboembolism, clinical decision support tools, thromboprophylaxis, hospitalized patients, electronic health records

## Abstract

Thromboprophylaxis of hospitalized patients at risk of venous thromboembolism (VTE) presents challenges owing to patient heterogeneity and lack of adoption of evidence-based methods. Intuitive practices for thromboprophylaxis have resulted in many patients being inappropriately prophylaxed. We conducted a narrative review summarizing system-wide thromboprophylaxis interventions in hospitalized patients. Multiple interventions for thromboprophylaxis have been tested, including multifaceted approaches such as national VTE prevention programs with audits, pre-printed order entry, passive alerts (either human or electronic), and more recently, the use of active clinical decision support (CDS) tools incorporated into electronic health records (EHRs). Multifaceted health-system and order entry interventions have shown mixed results in their ability to increase appropriate thromboprophylaxis and reduce VTE unless mandated through a national VTE prevention program, though the latter approach is potentially costly and effort- and time-dependent. Studies utilizing passive human or electronic alerts have also shown mixed results in increasing appropriate thromboprophylaxis and reducing VTE. Recently, a universal cloud-based and EHR-agnostic CDS VTE tool incorporating a validated VTE risk score revealed high adoption and effectiveness in increasing appropriate thromboprophylaxis and reducing major thromboembolism. Active CDS tools hold promise in improving appropriate thromboprophylaxis, especially with further refinement and widespread implementation within various EHRs and clinical workflows.

## 1. Introduction

Venous thromboembolism (VTE), encompassing deep vein thrombosis (DVT) and pulmonary embolism (PE), is the third most common cause of cardiovascular mortality [1] and substantially increases morbidity worldwide [2]. Approximately 60% of all VTE events are associated with a recent hospital admission, with the majority of VTE events—including most fatal PE—occurring in medical inpatients [3]. At the same time, VTE is associated with increased healthcare expenditure, especially in U.S. healthcare systems [4,5]. 

Thromboprophylaxis of hospitalized patients necessitates complex clinical management strategies that incorporate both patient-specific and disease-specific risk factors [6]. VTE risk models in surgical and medical patients have now undergone extensive external validation, including the Caprini VTE risk score in surgical patients and the Padua, IMPROVE, and IMPROVE-DD VTE scores in medical patients [7,8,9]. However, decreased average hospital length of stay, especially among medical inpatients (4–5 days) both in the U.S. and other advanced health systems has dampened the treatment effects of in-hospital thromboprophylaxis [8,10], with <4% of hospitalized patients receiving any form of post-discharge thromboprophylaxis [11]. 

Although antithrombotic clinical practice guidelines have given clear recommendations on the need for anticoagulant thromboprophylaxis in at-VTE-risk hospitalized patients [3,6,12,13], current hospital-wide thromboprophylaxis using systematic and evidence-based approaches remains sub-par [7,14,15,16,17]. Factors for the underuse of evidence-based approaches include the complexity of appropriate clinical management, the lack of incorporation of validated VTE risk models into clinical care pathways [18], and both the underutilization of thromboprophylaxis in high-VTE-risk patients due to concerns of bleeding and the overutilization of thromboprophylaxis in low-VTE-risk patients in the absence of formal VTE risk stratification [18,19]. 

Multiple approaches to improve the uptake of appropriate thromboprophylaxis in hospitalized patients have been assessed as follows: quality improvement programs that include educational activities and audits, national VTE prevention programs [20,21,22], passive human or electronic alert systems using clinical rules [23,24]; and more recently, active and computerized clinical decision support (CDS) tools [25]. CDS rules and algorithms include digital, paper-based, or in-person modalities or multimodal approaches [20,21]. More recently, a cloud-based and EHR-agnostic universal CDS tool incorporating a validated VTE risk model in hospitalized medical patients has been developed [26].

This narrative review will discuss various system-wide thromboprophylaxis interventions for hospitalized patients, with a focus on cross-platform CDS tools embedded into EHRs. This review will also outline evolving patterns in the literature that contribute to shaping future iterations of these tools, highlighting practices that are evidence-based and patient-centered.

## 2. Methods

Literature searches of the MEDLINE database were iteratively undertaken during the initial drafting of this paper, looking for relevant articles published between 1990 and 2023 with the following keywords and MeSH terms: “venous thromboembolism”, “thromboprophylaxis”, “risk assessment”, “clinical decision support”, “computerized systems”, “human alerts”, “paper-based systems”, “quality improvement”, “hospitalized patients”, and “major bleeding”. Observational studies and randomized controlled trials on the topic of a system-of-care approach to VTE prevention in hospitalized patients were included, as were meta-analyses and systematic reviews. Observational studies with fewer than 500 patients and randomized controlled trials with fewer than 100 patients were excluded.

## 3. Results

As shown in Table 1, there were 26 studies that met our criteria across the following four types of system-wide approaches for thromboprophylaxis in hospitalized patients: multifaceted interventions, the use of pre-printed order sets, passive alerts (human or electronic), and active CDS tools. 

### 3.1. Multifaceted Interventions 

The term multifaceted has been used to refer to an intervention combining most or all of the following components: local quality improvement efforts, practice guideline and clinical algorithm derivation, adaptation and/or dissemination, local audit and feedback provision at the team or individual level, and some optional complementary components that differ between studies. 

The earliest study of this paradigm compared usual care (A) and local-data-enriched education alone (B) and in combination with an audit and feedback component (C) [27]. The feedback component consisted of retrospective chart review finding reports at staff meetings and individual physician compliance feedback. Multivariate adjusted odds ratios (OR) compared to the control were 2.1 (95% CI, 1.6–2.9) for A, 3.6 (95% CI, 2.7–4.7) for B, and 3.8 (95% CI, 2.9–5.0) for C. In a study comparing an approach similar to (C) directed at physicians only versus at physicians and nurses, no significant differences were found regarding radiology-verified DVT (OR 1.21; 95% CI, 0.70–2.11; *p* = 0.50) [29]. The addition of sticker alerts and standing orders to the original study’s intervention was also associated with non-significant changes in appropriate thromboprophylaxis, as shown in a study of a wider intervention targeted at improving various clinical outcomes including VTE (The Stroke Practice Improvement Network) [30].

The SENTRY pilot cluster randomized trial added printed VTE risk assessment forms instead [31]. No significant difference was found in the appropriate thromboprophylaxis rates or the over- or under-prescription of thromboprophylaxis. The study also incorporated qualitative analysis components, including small interviews and/or questionnaires directed toward all stakeholders. 

Streubel et al. used prospective audits and bimonthly local VTE prevention guideline adherence rate reporting for patients undergoing primary total hip replacement surgery. They compared a historic control period to prospective audits and bimonthly presentations regarding VTE prophylaxis protocol adherence [28]. Compared with a historic control cohort, guideline adherence failure was lower (1.6% vs. 15%, *p* = 0.002) and VTE rates were similar (*p* = 0.37). 

Software infrastructure and hospital policy barriers prevented 11 of the 13 centers in the broad intervention implemented by the PREVENU study group from implementing a CDS tool as initially planned, meaning that the results were likely associated with the educational component [33]. No difference was found between the intervention and historic control cohorts in terms of thromboembolic events, major bleeding, all-cause mortality, appropriate thromboprophylaxis, or the composite of VTE and major bleeding. Multivariable analysis showed that the adjusted difference in the rates of thromboprophylaxis favored the intervention group by 6.6% [95% CI 1.6–11.6%]. 

A less specific intervention, not exclusively focused on VTE prevention, had similarly negative results: the Writing Group for the CHECKLIST-ICU Investigators and the Brazilian Research in Intensive Care Network (BRICNet). Investigators implemented a daily intensive care unit (ICU) checklist including a single item on whether thromboprophylaxis had been ordered or not. This was combined with daily rounds discussions, review of patient goals of care, and checklist adherence feedback [32]. Periodic text message reminders to use the checklist were sent to providers, and directors were contacted if adherence was low. VTE prophylaxis rates were not affected (74.8% vs. 75.0% of patient days; adjusted RR, 1.05; 95% CI, 0.91–1.22; *p* = 0.50).

The National VTE Prevention Program was developed in two pilot centers in the U.K. National Health System (NHS) and afterward expanded to all NHS centers in 2010 [22], combining educational programs, quality standards, and reporting requirements with audits, localization initiatives, and financial incentives. The national VTE risk assessment tool used included lists of patient- and admission-related risk factors for VTE and bleeding, but it was not validated. Hospitals were fined for not reaching the 95% screening rate target. Hospital-level data were collated and reported on a digital platform monthly and assessed on a quarterly basis, and root cause analyses were performed for a locally specified number of cases. The program resulted in a sustained increase in risk assessment rates by almost 50 percentage points in the first two years. Hospitals converged in terms of risk assessment rates from 51% (interquartile range (IQR) 27–71%) to 93% (IQR 91–96%). Hospitals achieving >90% risk assessment rates had 15% lower hospital-associated thrombosis, a 12% lower chance of avoidable VTE, and related mortality up to 90 days post-discharge. Non-fatal VTE readmissions and inpatient VTE-related mortality remained unchanged. 

### 3.2. Preprinted Order Sets 

An intervention by Fontaine et al. was informed by evidence-based, locally compiled prescription guidelines [34]. Physicians filled in anonymous questionnaires with data including patient characteristics, VTE risk factors, physician clinical impressions, and a visual analog scale for rating the risk of hemorrhagic complications. Over-prophylaxis decreased by 44% (from 25% to 15%) in the intervention group and increased by 17% (from 22% to 26%) in the control group. Appropriate thromboprophylaxis rates were not affected, while undertreatment showed minor reductions in both groups. The number of VTE risk factors was a significant factor affecting thromboprophylaxis prescriptions. 

### 3.3. Passive Alerts (Human or Electronic)

Passive alerts denote interventions that conclude with an alert and do not involve sophisticated risk stratification or significant clinician or patient data input. In contrast to the more complex and active clinical decision support systems mentioned below, they are not deeply integrated and have minimal automation. 

In a landmark study, Kucher et al. devised a computer program to perform daily automatic patient screening and scoring using ICD-9 codes and a weighted VTE scoring system [23]. For intervention patients with a score ≥ 4 not on thromboprophylaxis, physicians received alerts and a list of generic prophylaxis options, and VTE guidelines were made available on the screen. DVT or PE at 90 days occurred in 4.9% of the intervention group vs. 8.2% of the control group (*p* < 0.001). There was a 41% reduced risk of VTE at 90 days (hazard ratio 0.59, 95% CI, 0.43 to 0.81; *p* = 0.001). No differences were found in mortality or hemorrhage.

Another relevant study by Piazza et al. implemented manual weighted screening for VTE risk factors based on ICD-9 codes and laboratory values, using the same risk scoring system as Kucher [24]. Thromboprophylaxis contraindications and bleeding risk were not explicitly included. No specific thromboprophylaxis recommendations were made. Appropriate thromboprophylaxis rates increased across study periods in all hospitals and services from 27.1% to 51.9% (*p* < 0.01). There was no difference in overall VTE incidence or bleeding; however, there were significant reductions in DVT and overall VTE (*p* < 0.01), with an increase in PE (0.52 to 0.74, *p* < 0.01).

In a follow-up 2013 study, Piazza et al. focused on **discharge thromboprophylaxis**, with the manual screening of medical inpatients [37]. Staff were tasked with paging alerts and called attending physicians of high-VTE-risk patients to promote thromboprophylaxis. Contraindications, bleeding risk assessment, and specific thromboprophylaxis regimen recommendations were not provided. Thromboprophylaxis rates were significantly higher in the intervention group (22.0% vs. 9.7%, *p* < 0.0001), as were the rates of pharmacologic prophylaxis (19% vs. 7.7%, *p* < 0.0001). Symptomatic DVT or PE rates at 90 days were not significantly higher (HR 1.12; 95% CI 0.74–1.69). Mortality rates and bleeding rates at 90 days were similar.

Garcia et al. implemented a pharmacist manual chart review process based on a standardized and scored VTE risk assessment form, with weighted comorbidities/risk factors, contraindications, and prophylaxis options [36]. Physicians were only contacted once for each patient with scores of 4 or more. They were provided with information on VTE risk level, without specific prescription recommendations. The difference in thromboprophylaxis rates between the two groups was not statistically significant (*p* = 0.15).

Mahan et al. also tested an intervention based on manual clinical pharmacist VTE risk assessments and alerts, combined with rounds discussions and monthly performance reviews [5]. Printed risk assessment forms with risk factors and contraindications, plus prophylaxis recommendations based on bleeding risk and estimated glomerular filtration rate (eGFR) were used by the screening staff. In cases of physician non-compliance, lead pharmacists and physician champions were alerted. Appropriate prophylaxis rates increased significantly for critical care (OR 2.5), surgical (OR 1.6), and medical (OR 2.1) patients and overall discharges (OR 1.8) (*p* < 0.0001 for all). There was a 74% reduction in preventable VTE rates (*p* = 0.0006).

An early computerized alerting tool integrated with EHR order entry was implemented by Dexter et al. [35]. The tool provided physicians with reminders and prewritten orders with explanatory text regarding multiple preventive measures, to be accepted or rejected. Datapoints used in patient screening for the alerts included basic patient demographics, EHR codes denoting at least one indication, no contraindications, and no existing prescriptions. Measures were employed to capture user attention (e.g., the use of high-contrast color schemes and disabling the escape key). Subcutaneous heparin ordering for eligible patients increased from 18.9% to 32.2% percent (*p* < 0.001). 

### 3.4. Computerized CDS System (CDSS) Interventions 

Active CDS tools designed to aid with risk assessment and thromboprophylaxis prescription via EHR integrations represent a more sophisticated system-wide intervention for thromboprophylaxis and have been studied in both observational studies and, more recently randomized trials [26,42,46]. 

Early studies utilized non-validated, though sometimes weighted, scoring systems [39,40,41,42,43]. Galanter et al., in one of the earliest studies of this type, implemented a calculator [38], while most others were designed to display risk factors and corresponding risk strata, with the goal of acting as an aid to clinical judgment. Later studies, in contrast, utilized validated and weighted VTE risk assessment models in the form of calculators [26,44,46,47]. 

CDS tools were active, meaning they were designed to actively integrate information across the EHR and into the medication order screen, while other passive tools would simply provide written suggestions [40], and still, others would provide a generic order entry screen with prophylactic options for at-risk patients based on clinical service [39,40,41,42,47]. Some CDS tools had the capacity to cross-check existing orders to prevent double prescriptions and would verify patient characteristics beforehand to prevent the prescription of low-molecular-weightheparin(LMWH) for patients with a low eGFR. The more active CDS tools would suggest specific thromboprophylaxis regimens based on algorithmically defined processes of different levels of VTE risk [26,44,46,47]. The complexity of specific thromboprophylaxis regimens was also variable, with some individualizing LMWH doses only [44], while others would offer a wide array of options, including direct oral anticoagulants [26]. Finally, most of the CDS tools were integrated into order entry screens, whether generic or at admission, with notable exceptions [26,43,44,46]. Spyropoulos et al. differentiated a workflow based on triggering the discharge medication reconciliation screen, given the focus on post-discharge thromboprophylaxis not seen in other studies [26]. 

Bhalla et al. tested simple order sets displaying thromboprophylaxis options (including contraindications, lack of indication, and mechanical prophylaxis choices alongside pharmacoprophylaxis) for medical patients, as denoted by admission codes [41]. Non-medicine services served as the control. Thromboprophylaxis ordering saw significant increases in both groups as follows: 32.7% relative change, *p* < 0.001 in medicine, and 4.4% *p*< 0.001 in non-medicine services. The incidence of hospital-associated thrombosis was reduced from 0.65% to 0.42% (*p* = 0.008) for medicine patients but changes were non-significant for patients on non-medicine services. Bleeding rates increased from 2.9 to 4.0% on medicine services (*p* < 0.001) and from 7.7% to 8.6% on non-medicine services (*p* = 0.043).

In an intervention similar to the one by Bhalla et al. [41] but with more frequent repeats, a CDS tool was developed and deployed after a lecture and a consensus meeting, adapting guidelines into a local protocol [43]. At patient admission and then every 48 h if no thromboprophylaxis was prescribed, the tool would launch to enable the physician to choose among three risk levels and indicate if thromboprophylaxis was contraindicated, aided by displayed information. Additionally, in a move toward automation and more active forms of CDS, each level of VTE risk was linked to appropriate unfractionated heparin (UFH) regimens that were automatically prescribed. Physicians could override the CDS tool recommendations by providing a written justification. Appropriate thromboprophylaxis rates increased from 46.2% to 57.9%, representing a significant 11.7% difference (95% CI: 3.2–20.3%, *p* = 0.01). The increase in appropriate VTE prophylaxis among surgical patients was not statistically significant.

Umscheid et al., in a quasi-experimental study, tested a similarly simple screen displaying 11 thrombotic risk factors that provided generic thromboprophylaxis options [42]. Choosing to decline thromboprophylaxis required explicit explanation by default (free text or multiple choice). Contraindications were displayed further down in the admission order entry screen, while the system also automatically prevented double anticoagulant prescriptions and LMWH prescriptions in patients with advanced renal disease, based on auto-calculated patient eGFR. Thromboprophylaxis rates increased from 27.1% in the first to 51.9% in the third and final study period (*p* < 0.01). Appropriate prophylaxis rates showed a similar increase (from 42.0% to 54.4%, *p* < 0.01), while VTE incidence and bleeding rates were unaffected.

Macauley et al. tested a simple interface for all hospitalized patients [39]. A VTE risk level (high, moderate, or low) was selected by the physician at admission order entry. Relevant risk factors and their weights for a VTE risk score were simply displayed along with their weights in list format to aid with the decision. The next screen provided choices for contraindications or generic order options (not filtered according to the risk assessment results). Rates of pharmacologic thromboprophylaxis increased from 26% to 34% post-intervention (*p* < 0.0001), with a 57% relative risk reduction in VTE (*p* < 0.02). In a very similar study by Mitchell et al., an extra CDS component to the admission note history and physical was added to mandate risk stratification of patients as low-, medium-, or high-risk for VTE [40]. The overall rate of appropriate pharmacologic prophylaxis increased from 42.8% to 60.0% (*p* < 0.001). The overall VTE rate was significantly lower in the intervention group (1.1% vs. 0.34%, *p* = 0.001), though DVT rate differences alone did not reach significance. There was a trend toward lower bleeding rates after a reminder was added (1.1% vs. 0.6%, *p* = 0.09).

Galanter et al., who conducted the earliest study of this type in our review, implemented an EHR VTE risk assessment calculator that would activate alerts on the order entry screen [38]. After cross-referencing with existing orders, risk-appropriate prophylaxis options were recommended. A second alert was sent to the treating physician’s EHR inbox and printed at the nurses’ station for batch review. Pharmacoprophylaxis rates increased from 25.9% to 36.8% (*p* < 0.0001), but the VTE rate reduction was significant only in medical patients (0.55% vs. 0.33%, *p* = 0.02, number needed to treat of 450). The odds ratio of thromboprophylaxis was higher post-intervention (OR 2.02, 95% CI 1.92–2.13) and bleeding rates remained unchanged. 

Amland et al. implemented thromboprophylaxis interventions in three steps across three distinct periods separated by a washout [45]. First, a nursing staff workflow standardized care components including thromboprophylaxis orders and interventions. Next, a CDS tool allowing physicians to complete VTE risk stratification with guideline-derived criteria and thromboprophylaxis options was deployed, though no further details on the intervention were given. Finally, an alert was added to the order entry screen that would activate for patients not assessed or at increased risk of VTE, given undetectable utilization in the first two periods. VTE risk assessment rates within 24 h from admission increased from 49.7% to 78.4%, and VTE rates per 1000 patient days were 0.954 at baseline and ultimately decreased to 0.434 in the alert period, 35% lower compared with the baseline (OR 0.65, CI 0.49–0.87, *p* = 0.0039). The likelihood of VTE per patient after the full intervention was 29% lower compared with the baseline (OR 0.71, 95% CI 0.55–0.93, *p* = 0.014). 

After 2015, studies implemented validated VTE risk scoring tools, though there has been no convergence on one specific validated VTE risk assessment model [26,44,46,47]. Eijgenraam et al. presented one of the first studies to incorporate the validated Padua VTE score and piloted a button on the first page of the EHR that would deploy a risk assessment form based on physician request [44]. The use of the CDS was neither mandatory nor linked to the ordering system, though it did suggest appropriate prophylaxis regimens. The included bleeding risk assessment model was not validated. Adherence to guidelines was similar before and after the intervention (59.4% in both cases), but under-prophylaxis decreased (OR 0.48, 95% CI: 0.18–1.30, *p* = 0.14), while over-prophylaxis increased (OR 1.66, 95% CI 0.74–3.73, *p* = 0.22). Overall, 12.5% of patients on whom the CDS tool was used did not receive the LMWH dose prescribed by the system. On a scale measuring how often non-adherence was due to patient preferences, ranging from 1 (“never”) to 5 (“very often”), physicians responded with an average of 2.4 (standard deviation [SD] 0.5). Physicians questioned the validity of CDS advice for complicated patients, including those with multiple comorbidities, as well as whether the CDS was evidence-based, while 40% thought that automated ordering would reduce errors.

A randomized trial by Spirk et al. compared usual practice with an EHR alert prompting physicians, 24 h after admission, to verify whether a patient was on or had indications for prophylactic anticoagulation [46]. If not, the Geneva VTE risk score was presented, additionally with some demographic data pre-populated. The alert would be repeated three times if dismissed. A score ≥ 3 led to anticoagulant thromboprophylaxis recommendations based on renal function and bleeding risk factors. Overall, 55.5% of intervention patients had inconsistent or absent score calculations and lower appropriate thromboprophylaxis rates than those with consistent scores (62.6% vs. 71.8%, *p* = 0.006). Alerts increased overall thromboprophylaxis prescriptions from 63.1% to 70.4%, *p* = 0.028. No difference was found in the rates of appropriate prophylaxis and inpatient mortality; the rates of inpatient thromboprophylaxis and over-, and under-prophylaxis; inpatient all-cause mortality; inpatient VTE rates; and bleeding requiring medical attention. 

Mathers et al. tested a single-issue EHR-integrated alert at admission, mandating risk assessment of surgical and medical patients with the validated surgical Caprini VTE risk assessment module [47]. If patients were classified as medium- or high-VTE-risk, a prophylactic intervention was required. The CDS tool could be overridden in cases of critical bleeding or coagulopathy. Overall rates of pharmacologic prophylaxis increased from 60% to 81.2% (*p* < 0.001), and the increase was significant both in medical (26.3% vs. 62.8%, *p* < 0.001) and surgical services (83.7% vs. 95.5% *p* < 0.001). Non-adherence to CDS-recommended pharmacologic prophylaxis was higher in medical patients, with 12.7% not receiving the doses ordered versus only 3.6% of surgical patients. Patient preference (57%), provider overrides (25%), and patient absence for procedures or tests (15%) were common reasons for missing doses. A multivariate regression showed that hospitalization after the CDS was deployed was associated with higher odds of receiving pharmacologic prophylaxis (OR 4.72, 95% CI 2.94–7.57), as was being admitted to a surgical service (OR 14.3, 95% CI 8.62–24.39). Requiring blood transfusions was associated with lower odds of pharmacologic prophylaxis using the tool (OR 0.28, 95% CI 0.12–0.63). 

A recent large cluster randomized trial—IMPROVE-DD—by Spyropoulos et al. in medical inpatients evaluated a cloud-based, EHR-agnostic CDS tool incorporating the validated and weighted IMPROVE-DD VTE score after multiple rounds of usability testing [26]. The tool was triggered at admission, VTE prophylaxis order entry, and at discharge medication reconciliation. A mostly auto-populating calculator stratified patients as low-, moderate-, and high-VTE-risk and actively guided prescribers to appropriate pharmacologic thromboprophylaxis, including extended post-discharge thromboprophylaxis in high-VTE-risk patients with a score ≥ 4. Overrides were available only for high-bleed-risk cases and non-medical inpatients. The tool adoption rate was 77.8%, leading to increased appropriate inpatient (OR: 1.52, 95% CI: 1.39 to 1.67, *p* < 0.001) and appropriate at-discharge extended thromboprophylaxis (OR: 1.93, 95% CI: 1.60–2.33, *p* < 0.001). At 30 days post-discharge, there were fewer venous (2.7% versus 3.3%, OR 0.80, 95% CI 0.64–1.00), arterial (0.25% versus 0.70%, OR 0.35, 95% CI 0.19–0.67), and total thromboembolisms (2.9% versus 4.0%, OR 0.71, 95% CI 0.58–0.88) at intervention hospitals. Major bleeding was rare and did not differ between groups. Mortality was higher at intervention hospitals (9.1% versus 7.0%, OR 1.32, 95% CI 1.15–1.53), which included more patients hospitalized with COVID-19. 

### 3.5. Systematic Reviews and Meta-Analyses 

An early Cochrane systematic review and meta-analysis of randomized trials and observational studies showed that multifaceted interventions were effective at increasing the rates of thromboprophylaxis (Risk Difference (RD) 95% CI: 0.17, 0.09–0.25) [48]. However, there was substantial heterogeneity among the included studies, and the four non-randomized studies reporting VTE or DVT risk showed no difference. There was a significant increase in patients receiving appropriate prophylaxis with educational interventions (RD 0.11 (95% CI 0.06 to 0.17), although assessment by non-randomized studies and the pooled effect showed no statistical significance. Alerts increased the rates of overall thromboprophylaxis based on four randomized trials (RD 0.13, 95% CI: 0.01, 0.25, I-squared = 94.9%) and five non-randomized studies (RD 0.09, 95% CI: −0.00–0.19, I-squared = 97.8%). Electronic alerts tended to be more effective than their preprinted counterparts. Appropriate thromboprophylaxis rates were also increased by alerts, based on 10 non-randomized studies (RD 0.18, 95% CI 0.12 to 0.24), though there was substantial heterogeneity. All interventions increased the rates of thromboprophylaxis, with multifaceted interventions combined with alerts showing the greatest effect size. Absolute differences were moderate (less than 20%), with the greatest effect size in non-academic settings. 

An updated Cochrane systematic review and meta-analysis by the same group included 11 randomized trials on interventions to increase appropriate inpatient thromboprophylaxis [49]. The studies investigated multifaceted interventions, preprinted orders, and alerts (human or electronic). Though there was substantial heterogeneity in patient populations, hospital settings, and alert types among the studies, alerts (human or computer) overall increased the proportion of patients who received appropriate thromboprophylaxis by 16% and decreased the relative risk of symptomatic VTE by 36%. Although multifaceted interventions increased the proportion of patients who received prophylaxis, they were found to be less effective than alert interventions. 

A recent systematic review and meta-analysis evaluated the impact of CDS tools versus routine care on VTE prophylaxis guideline adherence and VTE rates in hospitalized non-surgical patients [25]. CDS interventions resulted in significantly increased rates of appropriate prophylaxis based on three non-randomized studies (OR 1.69, 95% CI: 1.25–2.28, *p* = 0.001, I 2 = 59.3%, *p* = 0.085) and increased overall rates of pharmacologic prophylaxis, based on seven non-randomized studies (OR = 2.02, 95% CI: 1.66–2.45, *p* < 0.001; I2 = 97.1%, *p* < 0.001). CDS tool use was also associated with significantly decreased rates of VTE events based on three non-randomized studies (OR = 0.68, 95% CI: 0.54–0.85, *p* = 0.001, I2 = 31.5%, *p* = 0.211).

## 4. Critical Synthesis and Discussion 

System-wide interventions to increase appropriate thromboprophylaxis in hospitalized patients require multimodal quality improvement efforts. The totality of the reviewed literature suggests three critical components of an optimal intervention as follows: provider and patient education; VTE risk assessment using validated models; and active rather than passive electronic alerts. 

Education increases provider motivation, provides current information on best practices, and can be facilitated by computerized modules [50]. Adjunctive motivational interviewing by clinicians can mitigate patient hesitancy, especially for extended post-discharge thromboprophylaxis, where patients have greater control over medication management. 

Risk assessment modules, especially when externally validated, weighted, and scored, represent an important improvement over intuitive risk assessment strategies. Validated VTE risk models such as the Caprini score in surgical inpatients and the Padua or IMPROVE/IMPROVE-DD score in medical inpatients have increased appropriate inpatient thromboprophylaxis in at- or moderate-VTE-risk patients and, in the case of IMPROVE-DD, increased appropriate at discharge extended thromboprophylaxis in high-VTE-risk patients without a concomitant increase in major bleeding [26].

An active CDS tool has clear advantages over passive interventions, especially if capable of auto-populating both a VTE risk score as well as an order entry for thromboprophylaxis. Active CDS tools appear to be effective in both increasing appropriate thromboprophylaxis based on VTE risk level and reducing major thromboembolism [26]. 

Below, we summarize key takeaway points on each major intervention category explored in this review.

### 4.1. Multifaceted 

Providing passive information to clinicians through lectures, printed materials, or online resources has an overall negligible impact on thromboprophylaxis [27,29,42,43,51,52,53]. This holds true even if the information is available at the point of care [27] or during medication order entry [54]. This has partly been attributed to barriers like patient preferences and time restrictions [29]. Educational interventions can be more engaging and effective when organized into small groups in the form of “academic detailing” [55], when incorporating hospital-specific data [27] and targeting varied team member roles, and when repeated as teams turn over [29,51,56]. These interventions have led to increased rates of appropriate thromboprophylaxis by highlighting the best practices [57] and rates of over-prophylaxis [33]. 

Designating a site champion can increase the success of multifaceted quality interventions [27,29,51]. However, even under ideal circumstances, champion reinforcement may be ignored by busy providers [51] and, moreover, requiring providers to perform tasks like entering risk scores can be error-prone [58]. Reminders by staff members may also be easily ignored by physicians [51]. Nevertheless, showing staff thromboprophylaxis rate targets, auditing, and providing ward-level feedback may increase adoption and performance [27,55,59]. Audits are effective in increasing thromboprophylaxis but require major time and staff investment [59]. Lastly, education and consensus building are not effective unless audited [52,57]. Overall, multifaceted interventions may increase appropriate inpatient thromboprophylaxis, though they show mixed results in reducing adverse clinical outcomes and are time- and resource-intensive.

### 4.2. Preprinted Order Sets 

Preprinted VTE admission order sets have increased thromboprophylaxis rates in settings where baseline rates were low [60,61]. However, as with order sets for other conditions, voluntary order sets can be easily ignored by busy providers [31]. 

### 4.3. Passive Alerts (Human or Electronic) and Order Entry Components 

Passive alerts have been moderately useful interventions, originally as stickers on patient files [59], printed schedule alerts [62,63], and eventually, electronic alerts for when thromboprophylaxis orders are inconsistent with patient risk profiles [54]. EHR alerts used as a VTE risk assessment point for all inpatients via a checklist interface resulted only in marginal improvements in appropriate thromboprophylaxis [47]. The lack of efficacy was attributed to the Hawthorne effect [64]. Alert fatigue [46] and provider workarounds [44] diminish utilization and effectiveness. However, repeat alerts can increase thromboprophylaxis in high-risk patients compared with single alerts [65], and prompts for verification by additional staff can sustain these increases [66]. Moreover, VTE risk assessment of patients that were automatically categorized as low-risk resulted in better outcomes compared with non-validated, ad hoc, point-based scoring systems alone [39]. Daily risk stratification paired with an alerting system resulted in decreased thromboembolism rates [67]. Additionally, prepopulating the order entry with relevant choices encouraged use and increased effectiveness [66]. Overall, passive alerts, especially in electronic form, appear to be more effective than labor-intensive multifaceted interventions or preprinted order sets. Making alert responses and order entry tool use mandatory, and embedding alerts within a multifaceted approach, can improve outcomes [42,60]. 

### 4.4. Computerized CDS Systems 

Active CDS tools offer the greatest potential benefit among the reviewed thromboprophylaxis interventions. Tools that incorporated individual clinical data were tested initially by printing alerts on surgical schedules for patients undergoing high-risk procedures [62]. Subsequently, alerts were automatically displayed and order sets provided pre-populated thromboprophylaxis options, with CDS tools incorporating a validated risk model [25,26,35,39] Thus, computerized CDS tools that are active are the most promising in implementing evidence-based medicine at the point-of-care based on accepted antithrombotic guidelines [39]. A major limitation of initial electronic active CDS technology was that it was limited to individual healthcare centers within a single EHR because of health informatics technology support limitations, with the inability to export to other health system EHRs, thus impacting generalizability [68]. EHR-agnostic and cloud-based CDS tools may overcome these limitations [26,69]. The most recent large cluster randomized trial utilizing an EHR-agnostic and cloud-based platform for a CDS tool that incorporated a validated VTE risk score demonstrated effectiveness in reducing major thromboembolism for hospitalized medical patients [26]. The ability to export a particular CDS tool to multiple EHRs using an EHR-agnostic platform and the ability to further refine and adapt a CDS tool based on local workflow requirements holds promise in effectively providing system-wide, evidence-based recommendations for the thromboprophylaxis of hospitalized patients at the point-of-care. 

## 5. Future Directions

**Figure 1:**
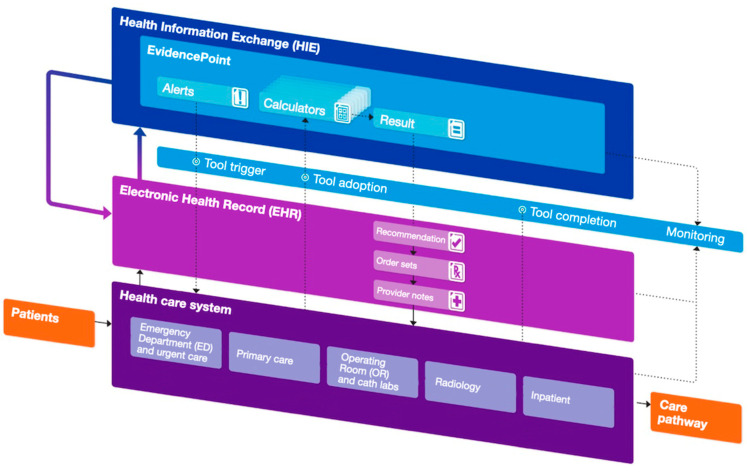
Schematics of the flow of data between the health care system, electronic health record (EHR), and health information exchange (HIE). Published with permission from [69]. Doctors launch an EvidencePoint clinical decision support tool from an EHR front-end workflow. The request includes the desired clinical prediction rule (e.g., the Well’s criteria) and the patient’s visit-specific ID. The clinical decision support tool forwards the request to the EvidencePoint application programming interface, which retrieves patient data, prepopulates evaluation answers, and sets the clinical prediction rule calculation logic. After calculating the patient score, the clinical decision support tool returns the score to the EHR front-end workflow.

**Figure 2:**
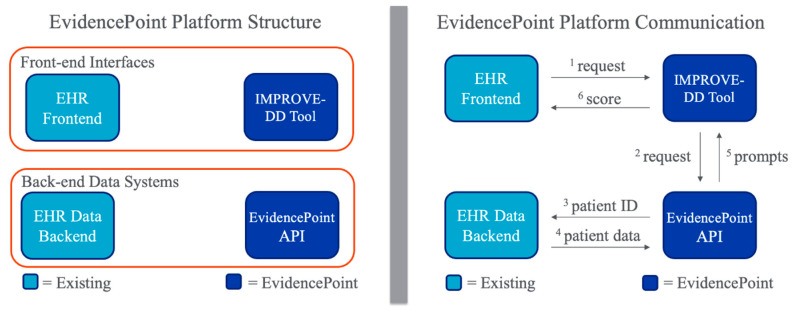
EvidencePoint platform structure (**left**) and communication scheme (**right**). API: application programming interface; CDS: clinical decision support; EHR: electronic health record. Users launch the CDS tool from a typical EHR workflow, or the tool is triggered automatically. The launch request includes the patient’s visit specific ID.^1^ The CDS tool forwards the request to the tool’s API,^2^ which retrieves the patient’s data from the EHR data backend^3, 4^ and pre-populates the tool with patient data where possible.^5^ The user fills in any remaining information and the tool calculates a personalized risk score for the patient, which is in turn sent back to the EHR^6^ to be incorporated into the patient’s medical record, as well as trigger any resulting next steps in the EHR, such as opening an order set.

The updated 2024 International Union of Angiology Consensus Guidelines on VTE prevention and management for the first time recommended the use of health informatics technology in the form of electronic alerts or CDS tools to identify key populations of medical inpatients that may benefit from inpatient and extended post-discharge pharmacologic thromboprophylaxis [70]. Antithrombotic guideline recommendations would thus encourage health system-wide adoption and implementation of validated CDS tools, and a recent National Institute of Health (NIH) R01 funding announcement calls for dissemination and implementation of validated CDS tools across multiple health-care environments (https://public.era.nih.gov, accessed on 22 February 2024). The previously discussed CDS platform—called EvidencePoint—utilized in the IMPROVE-DD trial is EHR-agnostic, thus theoretically interoperable within any EHR, and adaptable, thus able to be modified based on local usability testing to accommodate a variety of clinical workflows and health-care environments [69,71,72] (Figure 1). The platform could sit on top of any informatics infrastructure using internationally standardized SMART on FHIR and health level (HL) 7 applications [73]. CDS hook protocols can integrate with informatics systems to access information not only within the EHR but also across the health system informatics exchange environment. Single sign-on functionality can eliminate barriers that discourage adoption. Data are not only retrieved but also updated and created with minimal need for bespoke software. Importantly, this cloud-based EHR-agnostic CDS tool built as an online service is able to solve the tension between deep EHR integration and portability by using standardized secure protocols and application programming interfaces to pull and push data irrespective of the underlying informatics environment. The platform is able to retrieve data that auto-populate a specific VTE risk score and assist providers in determining VTE risk at the point of care, as shown in Figure 2 [69]. Thus, the portability and deep integration of the CDS tool would allow for flexible and seamless workflow integration into clinical care pathways utilizing formal usability testing with rapid iteration over designs, based on standardized metrics and feedback gathered through live testing sessions and interviews with volunteer clinicians. The flexibility of the platform would be in accordance with the “five rights” directive: the right information, to the right person, in the right format, through the right channel, at the right time [74]. For example, given the need for improved post-discharge thromboprophylaxis in high-VTE-risk patients, adding a discharge trigger point would require minimal effort.

Future research efforts for system-wide thromboprophylaxis interventions should focus on comprehensive quantitative and qualitative assessments of active CDS tools that have shown effectiveness and incorporate validated VTE risk score implementations, workflow components, and patient outcomes. Widespread refinement and implementation of active CDS tools across various EHR environments using EHR-agnostic platforms, with workflow mapping and usability testing of the CDS tools in diverse sites and in different clinical settings, will allow for the identification of barriers and opportunities for improvement. Lastly, there is potential for the use of artificial intelligence-based CDS using machine learning to increase CDS tool accuracy and improve model discrimination, which can lead to fewer false alerts and missed patients [75]. Rapid dissemination of effective, accurate, and adaptable CDS tools will enable the incorporation of current evidence at the point of care and thus promote thromboprophylaxis standardization across hospitals and health systems.

## 6. Conclusions

The system-wide implementation of thromboprophylaxis in hospitalized patients includes the use of multifaceted approaches with educational components and audits, the use of pre-printed order sheets, the use of human or electronic passive alerts, and, more recently, the use of active computerized CDS tools embedded within EHRs. Multifaceted and order entry interventions have shown mixed results in their ability to increase appropriate thromboprophylaxis and reduce VTE unless mandated through a national VTE prevention program. However, this approach cannot be easily exported to other health systems and is potentially costly and effort- and time-dependent. Studies utilizing passive human or electronic alerts have also shown mixed results in increasing appropriate thromboprophylaxis and reducing VTE. Active CDS tools have more consistently shown effectiveness in increasing appropriate thromboprophylaxis in hospitalized patients. Recently, a universal cloud-based and EHR-agnostic CDS VTE tool incorporating a validated VTE risk score revealed high adoption and effectiveness in increasing appropriate thromboprophylaxis and reducing major thromboembolism in medical inpatients. The refinement of effective CDS tools incorporating validated VTE risk scores with usability testing across various workflows that are deeply integrated across EHRs using agnostic methods, with widespread implementation of these tools, can potentiate the dissemination of best practices of evidence-based thromboprophylaxis in hospitalized patients. 

## Figures and Tables

**Table 1:** Summary of the study characteristics of the four system-wide thromboprophylaxis intervention categories for hospitalized patients at risk of venous thromboembolism.

Study	Study Type	Population	Intervention	Control	Risk Model	Outcome
Multifaceted						
Anderson 1994 N = 798 [27]	Cluster RCT	Medical and surgical	CME only: grand rounds slide lecture with local data by physician expert + mailed materials + telephone line for immediate VTE consults. CME + QA: additionally, data from manual retrospective chart reviews reported at medical staff meetings, individual physician feedback concerning compliance.	Usual care	Generic screening for the selection of high-risk patients and adequate thromboprophylaxis rates. Generic risk factor screening.	Changes in prophylaxis among the control, CME, and CME + QA hospitals maintained significance. Multivariate adjusted odds ratios were 2.1 (95% confidence limits, 1.6, 2.9) for control hospitals, 3.6 (95% confidence limits, 2.7, 4.7) for CME hospitals, and 3.8 (95% confidence limits, 2.9, 5.0) for CME+QA hospitals.
Streubel 2009 N = 345 [28]	Pretest–post-test	Surgical	Prospective VTE protocol adherence and event rate monitoring. Results presented every two months.	Usual care	N/A	VTE prophylaxis adherence failure reduction: 15% vs. 1.6% (*p* = 0.002), VTE rate trend for improvement (*p* = 0.37).
Labarere 2007 N = 812 [29]	Cluster RCT	Medical	Educational presentations with local data, educational material, audit/feedback components directed at physicians and nurses.	Physicians only	Evidence-derived, non-validated	No significant differences in radiology-verified DVT (two-level OR, 1.21; 95% CI, 0.70–2.11; *p* = 0.50; intra-cluster correlation coefficient, 0.08).
Hinchey 2010 N = 2071 [30]	Cluster, quasi-RCT	Medical	Audit, feedback, and benchmark information along with site-selected components from the following: evidence synthesis as educational resource, alerts, standing orders, grand round reporting of audits, individualized clinician feedback. Knowledge and attitude, barrier to adherence audit and discussion, with suggestions for improvement.	Audit with feedback	Unknown	Nonsignificant trend for appropriate thromboprophylaxis—no more data available. Extracted from figure with web plot digitizer (apps.automeris.io/wpd/): Intervention baseline 79%, outcome 87.1%. Control: baseline, 81%utcome, 86%.
Pai 2013 N = 2611 [31]	Pilot cluster RCT	Medical	Paper-based VTE risk assessment forms, educational sessions and material for all staff, real-time chart audits within 24 h of admission used for instructional feedback at 4, 12, and 16 weeks.	Usual care	Non-validated, evidence-derived ACCP 8th ed.	No significant difference in appropriate thromboprophylaxis, over- or under-prescription rates. Significant qualitative components: interviews and questionnaires of stakeholders, including patients. No significant difference rates of appropriate thromboprophylaxis between groups was found (OR = 0.80; 95% CI: 0.50, 1.28; *p* = 0.36).
Cavalcanti 2016 N = 6761 [32]	Cluster RCT	Medical and surgical	Goals of care discussions at the ICU level. Daily checklist in daily grand rounds with single item confirming thromboprophylaxis orders. Online and offline education. Involvement of the whole clinical team. Checklist adherence feedback. Periodic text message reminders. Directors contacted when adherence was low.	Usual care	N/A	VTE prophylaxis rates 74.8% vs. 75.0% of patient-days; adjusted RR, 1.05; 95% CI, 0.91–1.22; *p* = 0.5 favoring intervention.
Roy 2016 N = 15,351 [33]	Cluster RCT	Medical	Educational lectures, educational resources. In a second phase, a CDSS with alerts incorporating a medical diagnosis code for risk stratification and tailored thromboprophylaxis suggestions used in only 2 of 13 centers.	Usual care	Non-validated	No difference in VTE/major bleeding composite, thromboembolic events, major bleeding, or all-cause mortality. Thromboprophylaxis rates post-intervention similar between groups. Adjusted difference in thromboprophylaxis rates: 6.6% [1.6–11.6] favoring intervention.
Roberts 2017 NHS Centers in UK [22]	Observational pretest–post-test	Medical and surgical	See text.	Usual care	Paper-based, non-validated, evidence-derived national tool- lists of risk factors	Median risk assessment rate 2010: 51% (IQR 27–71%), March 2012: 93% (IQR 91–96%). Hospitals with >90% assessment rate: 15% reduction in hospital-associated thrombosis, 12% lower avoidable VTE, VTE-related mortality reduction post-discharge: 15% for >3 days hospitalizations (95% CI 0.75–0.96), 39% for <3 days hospitalizations (CI 0.48–0.79), excluding outpatient. Ninety-day readmissions with VTE: 4% reduction, secondary VTE diagnosis: 9% reduction, mean mortality rate: 9% reduction, maintained at 8% less than 2012 estimates, long-term data unavailable.
Pre-Printed Order Sheets						
Fontaine et al. 2006 N = 719 [34]	Cluster RCT	Medical	Evidence-derived, locally compiled thromboprophylaxis prescription guidelines. Anonymous anticoagulant prescription forms including patient characteristics (age, sex, body weight, date of admission), presence or absence of venous thromboembolic risk factors, 10 cm visual analogical scale of the patient’s risk of anticoagulation and hemorrhagic complications risk.	Usual care	Non-validated, evidence-derived, weighted risk factor-based	Over-prophylaxis increased by 17% (from 22 to 26%) in the control group and decreased by 44% (from 25% to 15%) in the intervention group. Appropriate thromboprophylaxis rates were similar (around 63%) before and after the intervention. No differences in undertreatment, with both groups showing minor reductions.
Passive Alerts (Human or Electronic)						
Dexter 2001 N = 1326 [35]	Cluster RCT	Medical	EHR order entry CDS providing rule-based reminders and prewritten orders with explanatory text. Rules integrated the demographics, EHR codes, and pharmacy records to alert and provide decision support that could be accepted or not by the physician. Disabled escape key and attention-grabbing color schemes used to increase use. Simulated use test and provider interviews for design.	Usual care	Non-validated, evidence-derived	Appropriate LMWH ordering rates increased by 13.3% (18.9% vs. 32.2%) favoring intervention (*p* < 0.001).
Kucher 2005 N = 2506 [23]	Quasi-RCT	Medical and surgical	CDS integrated into database performing daily automatic patient screening. Physicians of at-risk patients not on prophylaxis would receive alerts that had to be acknowledged and provide a list of generic prophylaxis order options. No forcing components. VTE guidelines made available in the EHR.	VTE guidelines made available in the EHR	Weighted score based on common risk factors and lab results	VTE rates at 90 days favored intervention with a hazard ratio of 0.59 (95% CI, 0.43 to 0.81; *p* = 0.001). Mortality and bleeding rates were similar.
Garcia 2009 N = 140 [36]	Cluster, quasi-RCT	Medical	Pharmacist manual chart review via a standardized risk-assessment form. Weighted and scored list of comorbidities/risk factors, contraindications, and relevant prophylaxis options. Standardized script for informing physician of patient VTE risk level, no specific therapy recommendations. No further alerts after the first.	Usual care	Weighted score based on common risk factors and lab results	Thromboprophylaxis rates similar (*p* = 0.15). In at-risk patients: Low-dose unfractionated heparin use rate: 56%. Prophylactic dose enoxaparin use: 11%. Sequential Compression Device use: 64% in intervention vs. 50% in control group, often in combination with pharmacologic strategies.
Piazza 2009 N = 2493 [24]	RCT	Medical and surgical	Manual weighted screening for VTE risk factors based on ICD-9 codes and laboratory values by staff. Alerting physicians if at-risk patient had no prescription with a recommendation for mechanical prophylaxis. Contraindications and bleeding risk not considered. No specific modalities, agents, doses, frequencies, or durations were recommended.	Usual care	Weighted score based on common risk factors and lab results	Thromboprophylaxis rates increased: 25.35% (95% CI: 21.8–28.9%). No differences in hard outcomes overall or in high-risk subgroups.
Mahan 2011 N = 3525 [5]	Observational pretest–post-test	Medical and surgical	Rounds discussions, manual pharmacist VTE risk assessments, alerts to physicians, monthly performance reviews. Printed risk assessment forms assessing risk factors, overall risk level and contraindications, with prophylactic recommendations as follows: enoxaparin, UFH, mechanical prophylaxis, or none based on bleeding risk and eGFR. Risk assessment forms added to the patient records. In cases of non-compliance, contact was repeated by the lead pharmacist and then escalated to a physician champion.	Usual care	Paper-based, non-validated, guideline-derived risk assessment form	Appropriate prophylaxis rates: OR 2.5 (critical care), 1.6 (surgical), 2.1 (medical), 1.8 (overall discharges), *p* < 0.0001. Preventable VTE rate reduction: 74%, *p* = 0.0006. Overall VTE reduction: 44%, *p* = 0.0624.
Piazza 2013 N = 2513 [37]	RCT	Medical	Pre-discharge manual screening of medical inpatients close to discharge. Staff page alerts and calls to attending physicians of high-risk patients with no active thromboprophylaxis orders. Contraindications, bleeding risk assessment, and specific regimen recommendations not provided.	Usual care	Weighted score based on common risk factors and lab results	Intervention group thromboprophylaxis rates: 22.0% vs. control 9.7%, *p* < 0.0001. Pharmacoprophylaxis rates: Intervention 19% vs. control 7.7%, *p* < 0.0001. Symptomatic DVT/PE at 90 days: HR 1.12 (95% CI 0.74–1.69), not significantly different. Mortality and bleeding rates at 90 days similar.
Computerized CDSS						
Galanter 2010 N = 38,647 [38]	Observational pretest–post-test	Medical and surgical	Evidence-based, locally compiled, EHR-integrated mandatory VTE risk assessment form, launched via alert at order entry until risk assessment completed. Could be dismissed for first 8 h. Form adapted based on previous answers and provided prophylaxis recommendations by risk level. Batch reviews of alerts printed at nursing station if patients were found to be at risk and had no valid orders based on automatic screening. Same alerts also sent to the clinical EHR mailbox of treating physicians.	Usual care	Evidence-based, locally compiled, non-validated	VTE pharmacoprophylaxis rate increased from 25.9% to 36.8% (*p* < 0.0001). Orthopedics only saw no increase. Intervention group prophylaxis rate higher for all medications except warfarin. Post-intervention odds of receiving prophylaxis: OR = 2.02, 95% CI = 1.92–2.13. Compared with medical patients, increased odds of prophylaxis for all patient types except obstetrics and gynecology. VTE rate declined from 0.51% to 0.43% (*p* = 0.22) Absolute VTE risk in medical patients declined from 0.55% to 0.33% (*p* = 0.02). NNT: 450 patients. Minor bleeding event rate post-CDS: 1.75% to 1.60% (*p* = 0.27).
MaCauely 2012 N = 4669 [39]	Observational pretest–post-test	Medical and surgical	Electronic admission order CDS. First screen: risk stratification as high, moderate, or low risk via point-based VTE risk assessment displayed as text along with relative and absolute pharmacoprophylaxis contraindications. Second screen: alert displayed for patients with moderate or high risk and no VTE prophylaxis. Option to order or indicate contraindication.	Usual care	Caprini surgical score	Post-implementation cohorts: Low-risk: 48%, moderate-risk: 31%, high-risk: 7%, higher manual risk classification than computer-generated: 38%, deferred/missing provider risk assessment: 14%, pharmacoprophylaxis rate from 27% to 53%, increase in VTE prophylaxis: 26% (*p* < 0.0001), VTE incidence declined from 0.98% to 0.42%, RRR 57%, *p* < 0.02)
Mitchell 2012 N = 5238 [40]	Observational pretest–post-test	Medical and surgical	Electronic alert in EHR history and physical note at admission. Asked whether patient is receiving prophylaxis and is low-, medium-, or high-risk for VTE. Displayed sample order choices for each level and listed contraindications. Note could not be saved without filling in the alert. Could not link to order screen because of software limitations.	Historical controls	None	Overall prophylaxis rate increased from 42.8% to 60.0%, *p* < 0.001. Not significant in renal failure, hip fracture/replacement patients. VTE rate decreased from 1.1% to 0.34%, *p* = 0.001. Non-significant DVT rate reduction from 0.42% to 0.13%, *p* = 0.053. Pulmonary embolism rate reduced from 0.74% to 0.22%, *p* = 0.009. Bleeding rate trend from 1.1% to 0.6%, *p* = 0.09.
Bhalla 2012 N = 36,500 [41]	Observational pretest–post-test	Medical and surgical	Mandatory VTE risk alert in admission EHR note including prophylaxis status, risk level. Sample orders for each risk category and contraindications displayed. Alert completion required to save note. Direct linking to order screen restricted by software limitations. Repeated every 5 days if no prophylaxis.	Usual care	None	(Medicine services) VTE prophylaxis order rates: 61.9% to 82.1%, *p* < 0.001, pharmacologic VTE prophylaxis rate: 59.0% to 74.5%, *p* < 0.001, hospital-acquired VTE incidence: 0.65% to 0.42%, *p* = 0.008, bleeding rates: 2.9% to 4.0%, *p* < 0.001. (Non-medicine services) VTE prophylaxis ordering rates: 70.5% to 73.6%, *p* < 0.001, pharmacologic prophylaxis rates: 59.3% to 63.3%, *p* < 0.001, bleeding rates: 7.7% to 8.6%, *p* = 0.043, hospital-acquired VTE incidence change nonsignificant.
Umscheid 2012 N = 223,062 [42]	Quasi-experimental pretest–post-test	Medical and surgical	EHR integrated CDS tools: list of 11 risk factors simply presented along with option to accept or decline VTE prophylaxis based on informed intuitive assessment, along with display of contraindications. Upon declining thromboprophylaxis, a specific reason had to be provided as free text in the first of two periods, changed to a choice of prewritten options during the third. The system disallowed two anticoagulants to be ordered simultaneously. An eGFR calculator prevented LMWH use in patients with stage 4 or higher renal disease. Risk estimation was intuitive.	Usual care	None	Thromboprophylaxis rates (control, first intervention period, second intervention period): 27.1% to 43.0% to 51.9%, *p* < 0.01. Appropriate thromboprophylaxis rates: 42.0% to 47.6% to 54.4%, *p* < 0.01. VTE incidence and bleeding rates: unchanged. DVT decrease: 1.77% to 1.75% to 1.15%, *p* < 0.01. Overall VTE decrease: 2.18 to 2.15 to 1.73, *p* < 0.01. PE incidence increase: 0.52 to 0.53 to 0.74, *p* < 0.01. Physician guideline adherence increase for positively predisposed: 89.0% to 93.8%, *p* < 0.01. Physician guideline adherence increase for not predisposed: 63.7% to 74.1%, *p* < 0.01. Non-compliance reasons: No risk factors 58%, on therapeutic anticoagulation 35%, peri-procedural concerns 4%, bleeding risk 2%.
Fuzinatto 2013 N = 523 [43]	Observational pretest–post-test	Medical and surgical	Educational lecture, consensus meeting, EHR-based CDS tool. At EHR launch every 48 h thereafter, if no thromboprophylaxis was prescribed, the physician could choose among three risk levels and indicate if thromboprophylaxis was contraindicated, aided by displayed text. Each level of risk was linked to appropriate UFH regimens, automatically prescribed in the background. Physicians could override the CDS by providing written justification.	Usual care	Evidence-based, locally compiled, non-validated	Thromboprophylaxis rate increase: from 46.2% to 57.9%, difference: 11.7% (95% CI: 3.2–20.3%, *p* = 0.01). Surgical patient VTE prophylaxis increase not statistically significant. Appropriate VTE prophylaxis pre- to post-implementation in cancer patients: 18.1% to 44.1%, absolute difference 26%, 95% CI: 9.9% to 42.3%, *p* = 0.002. Surgical patient postoperative appropriate VTE prophylaxis pre- to post-implementation: 53.6% to 60.4%, absolute difference 6.8%, 95% CI: −13.6% to 27.2%, *p* = 0.6. Medical patient appropriate VTE prophylaxis pre- to post-implementation: 44.2% to 57.2%, absolute difference 13%, 95% CI: 3.0% to 23.1%, *p* = 0.011.
Eijgenraam 2015 N = 128 [44]	Observational pretest–post-test	Medical	Button on first EHR, launching a risk assessment form, including a non-validated bleeding risk assessment model. Neither mandatory nor linked to the ordering system. Suggested appropriate prophylaxis regimens.	Usual care	Padua VTE risk score	Guideline adherence pre- and post-intervention: 59.4%, under-prophylaxis decrease: OR 0.48 (95% CI: 0.18–1.30, *p* = 0.14), over-prophylaxis increase: OR 1.66 (95% CI: 0.74–3.73, *p* = 0.22), CDS LMWH dose non-adherence: 12.5%, physician self-reported non-adherence reason mean, SD: 2.4/5, 0.5 due to patient preferences. CDS mistrusted for complicated cases by two/five physicians, three/five questioned the evidence base, four/five perceived improved patient outcomes, two/five believed automated ordering would reduce errors.
Amland 2015 N = 45,046 [45]	Observational pretest–post-test	Medical and surgical	Three distinct periods separated by washout as follows: 1. nursing staff workflow standardized (thromboprophylaxis orders, interventions, documentation, outcome tracking); 2. CDS tool for risk stratification, contraindication documentation, evidence-based recommendations; and 3. alert if patient not assessed or at increased VTE risk, given that initial tool utilization was non-measurable.	Usual care	Evidence-derived, non-validated risk assessment	VTE risk assessment rates within 24 h from admission: increased from 49.7% to 78.4%, percentage of at-risk patients identified: increased from 42.8% to 64%, at-risk patients prescribed thromboprophylaxis: increased from 25.4% to 47.7%, VTE rates per 1000 patient days at baseline: 0.954, after nursing intervention: 0.734, after CDS availability: 0.790, after alert implementation: 0.434 (55% lower than baseline), sustained VTE rate at study end: 0.407 per 1000 patient days. Full implementation reduced VTE prevalence from 0.36% to 0.17% (OR 0.65, 95% CI 0.49–0.87, *p* = 0.0039), likelihood of VTE per patient after full intervention 35% lower compared to baseline, alerts crucial for significant results.
Spirk 2017 N = 1593 [46]	RCT	Medical	EHR alert 24 h after admission prompting physicians to verify whether patient was on/had indications for therapeutic anticoagulation. EHR alert repeated at most three times prompted risk stratification. A few patient characteristics were prepopulated (e.g., age). Anticoagulation recommendations for LMWH, UFH, or mechanical prophylaxis based on creatinine clearance and bleeding risk, if appropriate based on risk.	Usual care	Geneva risk score	Similar rates of thromboprophylaxis, over- and under-prophylaxis, and hard outcomes; 55.5% with inconsistent risk assessment leading to 9.2% lower rates of appropriate prophylaxis (62.6% vs. 71.8%, *p* = 0.006).
Mathers 2017 N = 576 [47]	Observational pretest–post-test	Medical and surgical	Single-issue EHR-integrated alert at admission, mandating validated risk assessment. If patients were classified as medium or high risk, a thromboprophylaxis prescription was required. The CDS could be overridden in cases of critical bleed or coagulopathy (INR > 2).	Usual care	Caprini surgical score	Pharmacoprophylaxis overall rate increase: 60% to 81.2% (*p* < 0.001), medical service increase: 26.3% to 62.8% (*p* < 0.001), surgical service increase: 83.7% to 95.5% (*p* < 0.001), non-adherence in medical patients: 12.7%, non-adherence in surgical patients: 3.6%, common reasons for missing doses: patient preference (57%), provider overrides (25%), patient absence (15%), hospitalization post-CDS associated with higher pharmacoprophylaxis odds: OR 4.72 (95% CI 2.94–7.57), admission in surgical service associated with higher odds: OR 14.3 (95% CI 8.62–24.39), blood transfusions associated with lower pharmacoprophylaxis odds: OR 0.28 (95% CI 0.12–0.63).
Spyropoulos 2023 N = 10,699 [26]	Cluster RCT	Medical	EHR-agnostic CDS tool incorporating a validated VTE risk score for medical inpatient classification as low-, moderate-, and high-risk. Multiple trigger points (admission, VTE prophylaxis order entry, discharge medication reconciliation). Automatically populating risk score calculator. Directed prescribers to order-entry for appropriate pharmacologic thromboprophylaxis, including extended post-discharge thromboprophylaxis. System overrides available only for patients at high bleed risk and non-medical inpatients.	Education	IMPROVE-DD—validated for medical patients	Inpatient thromboprophylaxis rates increased: 80.1% vs. 72.5%, OR 1.52, 95% CI 1.39 to 1.67, *p* < 0.001. Appropriate discharge thromboprophylaxis rates in high-risk patients: 13.6% vs. 7.5%, OR 1.93, 95% CI 1.60–2.33, *p* < 0.001. VTE: 2.7% vs. 3.3%, OR 0.80, 95% CI 0.64–1.00, *p* = 0.048, ATE: 0.25% vs. 0.70%, OR 0.35, 95% CI 0.19–0.67 *p* < 0.001. Total TE: 2.9% vs. 4.0%, OR 0.71, 95% CI 0.58–0.88, *p* = 0.002. Major bleeding 0.15% vs. 0.22%, OR 0.69, 95% CI 0.28–1.69, *p* = 0.42. Mortality in the intervention group: 9.1% vs. 7.0%, OR 1.32, 95% CI 1.15–1.53 *p* < 0.001.

Abbreviations: CME: Continuing Medical Education, QA: Quality Assurance, N/A: Not Applicable, OR: odds ratio, CI: Confidence Intervals, ICD: International Classification of Diseases, RR: relative risk, CDSS: clinical decision support system, ACCP: American College of Chest Physicians, NHS: National Health Service, EHR: electronic health record, CDS: clinical decision support, LMWH: Low-Molecular-Weight Heparin, UFH: unfractionated heparin, RRR: relative risk reduction, eGFR: estimated glomerular filtration rate, INR: International Normalized Ratio, ATE: arterial thromboembolic event, VTE: venous thromboembolism, DVT: deep vein thrombosis, PE: pulmonary embolism.

## Data Availability

Data sharing is not applicable.
